# Enabling Real-World Data to Accelerate the Development of Innovative Cancer Biomarkers

**DOI:** 10.1055/s-0043-1768993

**Published:** 2023-05-23

**Authors:** Chen Yeh

**Affiliations:** 1 OncoDxRx, Arcadia, California, United States

**Keywords:** real-world data, cancer, biomarker, Dx, Rx

## Abstract

The molecular diagnostics industry has historically relied on sanitized clinical trials and commoditized data sources to inform its biomarker discovery and validation process—an under-substantiated approach that was ultra-expensive, resource-consuming and did not reflect how representative a new biomarker would be in broader patient populations. In an effort to gain more accurate insight into the patient experience and bring innovative biomarkers to market more efficiently and accurately, the industry is now expanding into extended real-world data. To access the needed breadth and depth of patient-centric data, diagnostic companies must collaborate with a healthcare data analytics partner that has three key assets: (i) a broad and deep megadata with metadata, (ii) a data-rich provider network, and (iii) an outcomes-improvement engine to support the next generation of molecular diagnostics (Dx) and therapeutics (Rx) development.

## Introduction

Precision medicine is the study of tailoring treatment regimens to account for a patient's genotype and phenotype. Precision medicine ultimately aims to generate and analyze data that can improve healthcare decisions. Any successful study of precision medicine involves developing a framework through which diseases, along with their severities, can be monitored. These efforts must also consider the dynamic nature of the human body. A patient's needs will change over time depending on their status, availability, and other factors. 1 2 Precision medicine is fast becoming a life-changing approach to the future of medical research. Patients respond differently to the same medications and have diverse phenotypes for the same disease. In turn, scientists have attempted to develop biomarkers for indicating disease states and responses to therapy. A biomarker is defined as a molecule that, when measured, indicates a normal biological process, a pathogenic state, or a response to an intervention or exposure. Biomarkers, combined with an understanding of disease mechanisms, would enable clinicians to provide patients the correct treatments early in their disease and thereby improve their clinical outcomes. Clinicians could also stratify patients before initiating treatment to ensure that patients receive the correct treatment. 3 At the heart of precision medicine, the overarching goal is to make sure every patient with cancer gets their best possible therapy. One critical approach to achieving this audacious goal is to develop innovative, highly quantitative, liquid biopsy cell-free DNA and cell-free messenger RNA based multivariate treatment selection and monitoring biomarkers that span therapeutic modalities. 4

In an ideal world, there would be time and resources to conduct large randomized controlled trials for every question in medicine. However, it is not generally ethical to randomize a late-stage cancer patient to placebo or a treatment with known and minimal benefit. Patients with cancer simply do not have time to wait for the ideal, especially when trial designs that can more efficiently provide reliable evidence for clinical utility are available. One emerging concept that is well-suited for biomarker development is a combined prospective–retrospective study, in which prospectively defined hypotheses are tested in archived specimens not collected specifically for that purpose. This is the method by which many widely used biomarkers for cancer therapy selection have been validated and shown to have clinical utility. For example, the association of wild-type but not mutated Kirsten rat sarcoma viral oncogene homolog (KRAS) with positive treatment response to anti-epidermal growth factor receptor monoclonal antibody therapies in colorectal cancer was based on retrospective analysis of samples from efficacy studies. 5 In another example, the pan-tumor approval for pembrolizumab based on tumor mutational burden was based on retrospective analysis of samples from the Keynote-158 study. 6 While the biomarker examples cited above relied upon studies conducted on samples collected during the course of controlled interventional trials, use of samples from well-designed observational cohort studies (whereby a group of patients are identified and followed over time for outcomes) can provide strong evidence for a given hypothesis. 7 Regardless of the source of samples, the key is to ensure that important design biases are avoided and/or accounted for so as to achieve the goal of demonstrating the biomarker and outcome association are true and not due to other unknown factors.

### Broader and Deeper RWD beyond EHR and Clinical Trials

In studies utilizing real-world data (RWD), multiple strategies should be leveraged to reduce and/or eliminate bias, for example, standardized eligibility criteria, which include the intended use of population for the biomarker under study; standardized procedures for sample collection, processing and shipment; a single laboratory processing all samples; harmonized data entry standards; and time-based outcomes anchored on a specific exposure (e.g., relevant medication start and post-sample collection) to better mimic the controlled start of an intervention as in a controlled trial. The real-world clinical–molecular database of uniformly collected test results and treatment outcomes is fueling new discoveries and new hope. By utilizing an efficient yet robust method for analysis of RWD, we are driving a paradigm shift in the treatment of people with cancer. We are expanding and optimizing the use of cancer therapies and delivering patients access to more options.

The industry (pharmaceutical, biotech, medical device, clinical communities) has invested significantly in data—specifically, extended RWD/real-world evidence (RWE). More importantly, the industry has realized that focusing on population health management (PHM) and outcomes improvement is its guiding principles and top goals, and big data are one key part of how it will achieve that goal. Patient-centric data beyond commoditized claims data assets is becoming instrumental in the biomarker validation pipeline. It is networking the process from discovery, new indications, clinical development, trial design, and measuring outcomes (e.g., side effects) to identify who is using an approved drug and why and determining value-effectiveness for drug reimbursement. As data has become a staple decision driver at most companies, organizations are increasingly aware of the need to bridge the gaps among these multichannel and multilayer data sources.

**Fig. 1:**
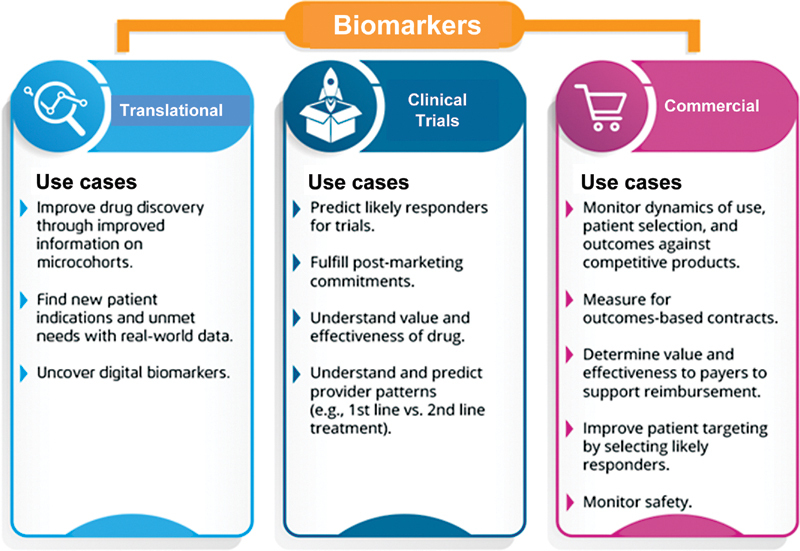
Leveraging extended real-world data/real-world evidence across the biomarker development cycle.

The biomarker development process takes 3 to 5 years, and relies on an expensive and often inefficient clinical trials process, as well as costly, sparse data that does not provide a full picture of patient health history. For example, claims data will show that a patient fills a prescription but gives no insight into outcomes, side effects, etc. Diagnostics (Dx) and therapeutics (Rx) companies can improve this process and save costs by partnering with a healthcare data analytics vendor to access and leverage extended RWD and RWE to better understand the targeted populations and their outcomes ( Fig. 1 ). As the entire system (regulators, payers, manufacturers, and providers) aligns around outcomes, high level of standardization will enable a fair, outcomes-driven healthcare system. Healthcare today has a crucial opportunity, as, for the first time, key industry players are aligning on the same key goals. Regulatory, cost, and reimbursement pressures are driving the urgency to deliver the right treatment to the right patient, as measured by real-world outcomes and monitoring. This means that manufacturers, payers, and providers all benefit from solving similar challenges, especially the targeted populations of lower risk and highest benefit; the management of overall balance between clinical and financial outcomes; the identification, selection, validation, and monitoring of relevant biomarkers; and the assurance of high standards of testing performance, training, education, support, and follow-up to maximize patient outcome.

The current guidelines aim to accelerate medical product development and innovation and place more focus on RWE- and RWD-driven decision making. By definition, RWE is data from sources other than clinical trials (e.g., randomized trials and observational studies) on the use and the potential benefits or risks of a biomarker; however, RWD is data on patient health status and delivery of care that's routinely collected from electronic health records (EHRs), claims and billing, patient-generated clinical data, and more. Both RWE and RWD are growing in volume and depth with the increasing use of computers, mobile devices, and wearables and gaining utility as advanced analytics capabilities (e.g., artificial intelligence and machine learning) enable more personalized and actionable insights. The U.S. Food and Drug Administration further detailed the usefulness of RWD in trials for new therapies. The emphasis on RWD is leading to a new approach that includes pragmatic trials (e.g., trials where the control arm is based on RWD from the standard of care) and synthetic cohorts (generating historical controls from historically accumulated trial controls, and/or simulating them on current RWD cohorts). Because pragmatic trials are poised to slash costs and reduce timelines drastically, companies that adopt them early will differentiate themselves in the market.

**Fig. 2:**
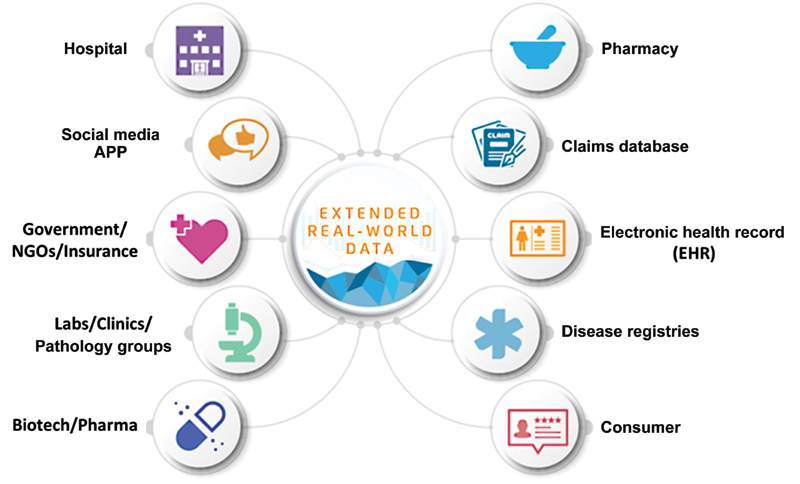
Extended real-world data from multichannel and multilayer healthcare sources. NGOs, nongovernment organizations.

Key RWD/RWE challenges are emerging around access to health systems and patients as different organizations compete to enroll patients in traditional studies, as well as innovative studies that leverage data-driven approaches. It is not sufficient for companies to leverage data; they must also create clear value for providers and patients, ensuring that innovations in clinical development help health systems achieve certain goals, for example, the development of population health and clinical operations strategies that align clinical trials with standards of care; and diligent selection of trials that are truly of value to the patient and to the health system that manages them. A real-world approach marries clinical development with the realities of healthcare—comorbidities, complexity, and the need to measure real-world outcomes (versus synthetic outcomes within a sanitized trial setting). By leveraging extended RWD/RWE, companies gain critical value across their pipeline, allowing them to shorten clinical trials time and time to market, to create a more insightful digital marketing strategy, to improve matching of patients with therapies, and to significantly reduce overall cost. Businesses have started to grow around certain therapeutic areas (e.g., oncology EHRs), and healthcare analytics vendors are now expanding services to meet the demand for integrated data from many sources (e.g., laboratories, consumer behavior, and more) that capture the breadth of patient health ( Fig. 2 ).

### Outcome Improvements Powered by Extended RWD

The industry historically placed its largest bets on heavily sanitized clinical trials, as regulatory approval equated to reimbursement. As the field evolved, developers learned that the synthetic trial setting did not reflect drug performance in the real world. When commercialized and available to larger populations, drugs may produce different safety profiles and effectiveness than in the select trial population. The limited populations and controlled nature of clinical trials have sparked the extended RWD/RWE movement to understand patient behavior and real-world performance via data outside the clinical trial setting. And as regulators and payers adapt, the pressures have mounted to develop therapies that are safe and effective in the real-world setting. Much of the initial evidence, however, was confined to large, but shallow, claims-derived datasets. These datasets present many challenges in the clinical trials setting, for example, coding challenges, data quality challenges, provenance tracing, and selection of outcomes metrics. Further, EHR-derived datasets emerged, especially for some diseases (e.g., cancer) from specialized companies or from specific regions, payers, etc. Most of these datasets, however, are not broad and deep enough and can often only be partially linked together.

Driving outcomes improvement requires integration of data across sources, but with much of RWD to date focused on claims and with limited EHR data, the industry lacks the breadth and depth to leverage RWD in biomarker and drug development. The industry needs to access 92% of data remaining outside of EHR to fully capture the patient experience. Having large claims data and some EHR data is only scratching the surface of true outcomes measurement and transformation work for both population health and personalized approaches. Specialty EHRs (e.g., oncology and cardiology) fill in some commercialized data gaps but miss a lot of the data that drives population health (e.g., cost, patient satisfaction and laboratory results). Extended RWD/RWE requires broader sources to truly understand patients; Dx and Rx companies can access this knowledge by partnering with an established healthcare data analytics provider. As data sources expand into patient-reported information, integrated vendor analytics will become more critical to round out extended RWD. Having RWD and RWE is only the start to driving outcomes; companies need the ability to act on the data by turning it into provider-level actions that benefit the patient. For example, a Rx company can use shallow data to predict drug response, find patterns, and develop insights, but only when it deploys the drug in the real-world setting can it understand the actual real-world impact.

## Conclusion

Despite their information-rich potential, collecting RWD and producing RWE require scientific vigilance. Without adhering to proper study design, real-world studies are unwittingly subject to design errors that can favor one treatment or device over others, leading investigators to inherently flawed inferences. To deliver on the promise of RWD, it is critical for all players to understand the impact of statistical biases caused by design errors. The core capabilities of best practice, analytics, and adoption that support outcomes improvement can also serve as an effective framework in biomarker and drug development. With a trusted network of RWD providers, companies can merge data and have unique dataset to quickly identify solutions, leverage technologies for data ingestion and visualization, and complement it with deep operational expertise to contextualize the human factors and processes that drive success. While the industry is accustomed to utilizing data for certain insights, its next step is to scale those insights into actions—similar to how regulators and payers have realized the value of extended RWD for key decisions concerning regulatory approvals and reimbursements. By leveraging extended RWD, real-world insights, trusted provider networks, PHM approaches, and the definitions and real-time measurements of real-world outcomes, Dx and Rx companies can achieve meaningful outcomes-driven approaches to the development, regulation, launch, reimbursement, and monitoring of new biomarkers and therapies. In today's digital world, the ubiquity of real-world data collection has created a wealth of data points from which companies can build RWE. There is increasing interest and potential for converting RWD into RWE that, through careful design, addressing bias, analysis, and interpretation, can be used to inform healthcare decision making. To help pave the way forward, initiatives and guidelines were established and updated with the aim to improve the transparency, reproducibility, and validity of real-world studies, ultimately leading to the improvement in patient outcome.
